# Effect of long-term deficit irrigation on tomato and goji berry quality: from fruit composition to *in vitro* bioaccessibility of carotenoids

**DOI:** 10.3389/fpls.2024.1339536

**Published:** 2024-01-24

**Authors:** Thomas Breniere, Anne-Laure Fanciullino, Doriane Dumont, Carine Le Bourvellec, Catherine Riva, Patrick Borel, Jean-François Landrier, Nadia Bertin

**Affiliations:** ^1^ INRAE, PSH UR1115, Avignon, France; ^2^ Aix-Marseille Université, INSERM, INRAE, C2VN, Marseille, France; ^3^ Avignon Université, UPR4278 LaPEC, Avignon, France; ^4^ Univ Angers, Institut Agro, INRAE, IRHS, SFR QUASAV, Angers, France; ^5^ INRAE, Avignon Université, UMR408 SQPOV, Avignon, France

**Keywords:** *Solanum lycopersicum*, *Lycium barbarum*, drought, quality, phenolic compounds, antioxidant, carotenoid bioaccessibility

## Abstract

Drought is a persistent challenge for horticulture, affecting various aspects of fruit development and ultimately fruit quality, but the effect on nutritional value has been under-investigated. Here, fruit quality was studied on six tomato genotypes and one goji cultivar under deficit irrigation (DI), from fruit composition to *in vitro* bioaccessibility of carotenoids. For both species, DI concentrated most health-related metabolites in fresh fruit. On a dry mass basis, DI increased total phenolic and sugar concentration, but had a negative or insignificant impact on fruit ascorbic acid, organic acid, and alcohol-insoluble matter contents. DI also reduced total carotenoids content in tomato (−18.7% on average), especially β-carotene (−32%), but not in goji berry DW (+15.5% and +19.6%, respectively). DI reduced the overall *in vitro* bioaccessibility of carotenoids to varying degrees depending on the compound and plant species. Consequently, mixed micelles produced by digestion of fruits subjected to DI contained either the same or lesser quantities of carotenoids, even though fresh fruits could contain similar or higher quantities. Thus, DI effects on fruit composition were species and genotype dependent, but an increase in the metabolite concentration did not necessarily translate into greater bioaccessibility potentially due to interactions with the fruit matrix

## 1 Introduction

Agriculture faces the dual context of climate change and human health, both of which are reflected in the “one health” concept. In particular, the increasing risk of more intense and frequent droughts and the issues caused by water scarcity pose a constant threat to global agricultural productivity and food security (Spinoni et al., 2018). As far as health is concerned, the consumption of fruit and fruit-derived food products is consistently described as a lever for both the protection and improvement of health (Poiroux-Gonord et al., 2010; Slavin and Lloyd, 2012). In this study, this dual context was approached in a cross-disciplinary manner, studying the impact of water resources on the quality of two species, tomato and goji, whose fruits potentially contribute to consumer health, and going beyond disciplinary studies by assessing quality in terms of composition as well as *in vitro* bioaccessibility of carotenoids, one of the main targets of health effects. Whereas tomato fruit (*Solanum lycopersicum*) is one of the most consumed fruit and vegetable products in several countries (Costa and Heuvelink, 2018), due to the diversity in its product forms (fresh, cooked, juice, concentrate, etc.), goji berries (*Lycium barbarum*) are often categorized as a “superfruit” due to their polysaccharide (dietary fibers) content and the richness of their phytochemical profile. Both fruit species contain large amounts of minerals, vitamins, and phenolics, which may overall independently or synergistically contribute to fruit health potential (Socaciu, 2007; Story et al., 2010; Amagase and Farnsworth, 2011). In particular, the large number of pigments in tomato and goji berry makes these fruits an interesting source of carotenoids (Socaciu, 2007; Story et al., 2010), which give the fruit their yellow, orange, and red colors and also have antioxidant properties; some are a source of provitamin A, supporting visual functions and mitigating the outcome of some types of cancer (Bohn et al., 2021).

Crops with high water consumption such as tomatoes grown for industrial processing, mainly in the Mediterranean regions, will be severely challenged by future environmental changes (Cammarano et al., 2022). The genetic diversity of tomato and the existing genetic resources offer a number of advantages for better adaptation to water deficit (Diouf et al., 2020). On the other hand, goji plants are known to adapt to arid climates (Wei et al., 2006), although the mechanisms responsible for this adaptation remain to be elucidated. The development of goji berry production could thus be of benefit in reducing dependency on irrigation and increasing the sustainability of health-promoting fruits. Beyond the predictable effects of water deficit on fruit yield, the question of the impact on quality also arises. It has been widely documented on tomato (Bertin and Génard, 2018). In particular, moderate deficit irrigation (DI) has been described as beneficial for the improvement of the nutritional quality, aroma, and carotenoid content of fruits from specific tomato varieties. It does so by stimulating the secondary metabolisms of plants (Ripoll et al., 2014; Coyago-Cruz et al., 2022), although much of the response is also influenced by genetic and seasonal factors, along with the intensity and duration of the DI treatment (Ripoll et al., 2014). More recently, data on the impact of environmental conditions on *L. barbarum* fruit quality traits are emerging, with recorded changes in fruit phenolic composition and carotenoid concentrations that depend on the harvest date (Poggioni et al., 2022). To our knowledge, the impact of DI on goji fruit quality traits remains unexplored.

In the context of a study on the health value of fruit, it seems essential to assess the bioaccessibility of target compounds beyond variations in their content in the fruit, which is rarely done in studies that report an effect of production conditions on fruit quality. Bioaccessibility and/or bioavailability data provide information on the release and assimilation of potentially beneficial compounds in the gastrointestinal tract during digestion, this being the first step for compound uptake and metabolism by the human body (Morelli and Rodriguez-Concepcion, 2023). Previous research has described how factors such as the food matrix or post-harvest treatment may affect carotenoid bioaccessibility (Van Het Hof et al., 2000; Borel, 2003), but as far as we know, there are no available data on how water deficit affects the bioaccessibility of carotenoids in fleshy fruits. Depending on the carotenoid chemical structure and fruit matrix, bio-fortification strategies to increase concentrations of phytochemicals in edible crop tissues may not necessarily translate into increased levels of incorporation into the intestinal micelles and hence into health benefits (Bassolino et al., 2022; Morelli and Rodriguez-Concepcion, 2023). The present work investigated the impact of DI on tomato and goji fruit quality traits with a focus on carotenoid content and bioaccessibility, which was assessed using an *in vitro* digestion model. By selecting different tomato genotypes with a well-defined genetic background associated with carotenoid heterogeneity, it was possible to evaluate the degree to which specific compounds are affected by DI, considering possible interactions between genotype and DI in the expression of desirable fruit traits. The comparison between tomato and goji allowed us to explore the hypothesis that the impact of drought on fruit quality may differ between species due to differences in these fruits’ matrices and carotenoid nature. Finally, the positive effect that DI is assumed to have on fruit quality was discussed considering variations in both the fruit composition and the carotenoid bioaccessibility.

## 2 Materials and methods

### 2.1 Genetic material

Four tomato introgression lines (ILs), IL2-5, IL5-4, IL6-2 and IL12-4, originating from a cross between cv. M82 and the wild green-fruited species *Solanum pennellii* (accession LA 716) (Eshed and Zamir, 1994), were selected for the variety of fruit physical and chemical properties (Rousseaux et al., 2005). All ILs are almost isogenic to the original M82 genotype with a single homozygous chromosome segment (location indicated in the name) incorporated from *S. pennellii* (Liu et al., 2003). M82, the reference genome, has been previously described as “drought sensitive” (Liu et al., 2017). IL2-5 is described as a “drought tolerant” mutant compared to M82 (Liu et al., 2018). IL5-4 is described as improved in terms of °Brix, reduced firmness, and sensitivity to blossom end rot (Matsumoto et al., 2021). IL6-2 contains a Beta mutant gene (B) that alters carotenoid biosynthesis, which leads to an increase in fruit β-carotene content at the expense of lycopene (Ronen et al., 2000). IL12-4 fruits contain higher levels of antioxidants, ascorbic acid, and soluble solids compared to M82 (Sacco et al., 2013). In addition, tomato cv. H1311 was selected for its high lycopene content (Ozminkowski, 2018).

One goji (*L. barbarum*) cultivar, SWEET CAROLINE “FPW07” (FPW Développement, France) was selected. It displays beneficial agronomic traits such as high yield and powdery mildew resistance (Wang, 2019).

### 2.2 Glasshouse experiment

Tomato seeds were sown in plug trays in a climatic plant growth chamber with 70% relative air humidity, 120 µmol m^−2^ s^−1^ photosynthetic photon flux intensity, 14 h artificial daylight, and day–night temperatures set at 22–17°C. Following germination, seedlings were transferred to a glasshouse and transplanted individually into 0.3-L pots, then, at the third true leaf stage, into 7.5-L pots filled with compost substrate made of 90% organic matter (2/3 frozen black peat moss, 1/3 peat moss) with a 80% water retention capacity (Potgrond h70 047, Klasmann-Deilmann France). Six blocks of two plants per genotype (*N* = 12 plants per genotype) were randomly distributed over six rows surrounded by two border rows. The day–night temperature set point was 25–17°C. Flowers were pollinated by bumblebees. Two-year-old goji plants (*N* = 60 plants) were grown into 7.5-L pots in the same growing conditions as tomato plants. The goji plants were homogenized in terms of ramifications per plant, following the three-level model and were then arranged on a trellis (Jiao and Liu, 2020). Flowers were self-pollinated.

The nutrient solution (Liquoplant Rose, dilution 4/1000, Plantin, Courthézon, France) was supplied to plants by a drip irrigation system (drip flow of 2 L h^−1^) for the whole growth period with an average electroconductivity of 1.8 mS cm^−1^, and a pH of 6.0. During the vegetative period, the number of daily triggers was revised periodically to match 100% replacement of plant evapotranspiration. When flowers became visible in 75% of plants, half of the rows (*N* = 3 blocks of two plants for each tomato genotype, *N* = 30 for goji) were assigned to one or other of the two irrigation regimes, namely, control irrigation (CI) and DI. CI plants were irrigated in the same manner as during the vegetative period. For DI plants, the water supply was gradually decreased until a 50% reduction of irrigation volume that was maintained over the whole reproductive period up to final fruit harvest (Djidonou et al., 2016). Soil water content was tracked using probes (*N* = 5 random plants per species per treatment) inserted below the soil surface (EC-5 Soil Moisture Sensor, Decagon). The average volumetric water content for the CI and DI time periods was around 45% (soil water potential about −0.1 MPa) and 25% (soil water potential about −0.2 MPa), respectively (**Supplementary Figure 1**). Over the whole fruit production period, water delivered to CI tomato plants ranged from 159 to 167 L plant^−1^, falling to 69–76 L plant^−1^ under DI. Total water delivery for goji was on average 190 L plant^−1^ for CI and 105 L plant^−1^ for DI.

### 2.3 Fruit harvest and biochemical analyses

Red-ripe fruits were collected twice a week during the harvest period, which lasted 48 days for tomato and 75 days for goji. Commercial fruit fresh yield (CFFY) was calculated by weighing all harvested mature fruits except for tomato fruits with blossom end rot. Average fruit weight was approximated by dividing CFFY by the total number of collected fruits. Calculation of commercial fruit dry yield (CFDY) was based on dry matter (DM) content assessed gravimetrically by drying a sub-sample at 75°C until weight stabilization. Water use efficiency was calculated on the basis of fresh (FW-WUE) or dry (DW-WUE) weight by dividing CFFY or CFDY by the total amount of water supplied in each treatment.

For the biochemical assessment of fruit quality, five (tomatoes) to six (goji berries) separate batches were formed for each treatment and genotype by mixing fruits (*n* > 30) harvested over the entire harvest period, in order to attenuate any seasonal changes in fruit quality (Poggioni et al., 2022). Each representative sample was grounded (IKA A11 basic; IKA-Werke GmbH, Germany) in liquid nitrogen and around 30 g of fresh powder was stored at −80°C in sealed containers. The biochemical analyses were performed on fresh powder (H-ORAC_FL_ and carotenoids) or on freeze-dried samples [alcohol-insoluble matter (AIM), sugars, acids, vitamin C, and phenolics].

AIM, as an estimation of fruit dietary fiber, was measured after extraction and removal of soluble molecules using 80% and 50% ethanol solutions, following starch hydrolysis by amyloglucosidase, as previously described (Gilbert et al., 2009).

Using the H-ORAC_FL_ assay, hydrophilic antioxidant capacity was measured. For this purpose, 0.5 g of thawed tomato or goji powder was diluted in 5 mL of pH 7.4 phosphate buffer and mixed in a sealed container at 160 rpm for 1 h. After centrifuging at 3,300 *g* for 10 min, 25 µL of the supernatant was added to a 96-well plate. One hundred fifty microliters of 0.5 nM fluorescein (FL) solution (Sigma Aldrich) was then added and incubated at 37°C for 10 min. Antioxidant capacity was gauged using fluorescence intensity at 485/520-nm wavelengths. After a 5-min stable phase, 25 µL of 150 mM AAPH [2,2’-azobis(2-amidinopropane)dihydrochloride] was added, starting the oxidation. Fluorescence was measured at 40-s intervals for 1 h, and values were compared to the initial stable phase reading. Results, derived from the fluorescence decay curve, were given in micromoles of Trolox equivalent (TE) per gram on a DM basis using a Trolox calibration curve (Huang et al., 2002). Three technical replicates were averaged for each genotype-treatment condition.

Soluble sugars (glucose, fructose, and sucrose) and organic acids (citric acid and malic acid) were extracted with a chloroform–methanol–water mixture (3:5:5 v:v:v) as described elsewhere (Gomez et al., 2002). Soluble sugars and organic acids were determined by HPLC, using a refractometer detector for soluble sugars (Gomez et al., 2002) and a UV detector for organic acids (Wu et al., 2002). In goji berry, with the chromatographic peak for citric acid being poorly resolved, this molecule was assayed using indirect enzymatic measurement of the disappearance of NADH by the action of citrate lyase, malate dehydrogenase, and lactate dehydrogenase with a microplate reader as described elsewhere (Wu et al., 2002).

Tomato carotenoids (β-carotene, lycopene, phytoene, and phytofluene) were extracted with a hexane-dichloromethane-ethyl acetate mixture (1:4:50 v:v:v) and then assayed by HPLC DAD as described elsewhere (Sérino et al., 2009). For goji berry, carotenoids (β-carotene, zeaxanthin dipalmitate, zeaxanthin, β-cryptoxanthin palmitate, and β-cryptoxanthin) were extracted with an ethanol–hexane mixture (2:1 v:v) then assayed by UPLC ESI TQ as described elsewhere (Dumont et al., 2020). No distinction was made between the E and Z forms of carotenes, and the mass of the peaks observed was integrated into a single molecule.

Tomato phenolic compounds (chlorogenic acid, cryptochlorogenic acid, rutin, and naringenin chalcone) were extracted with a methanol–water mixture (7:3 v:v) and then assayed by HPLC-DAD (Jordan et al., 2020). For goji berry (rutin, chlorogenic acid, and neochlorogenic acid), the protocol applied was a methanol–water–formic acid extraction (7.6:2:0.4 v:v:v) and an assay by UPLC ESI TQ as described elsewhere (Dumont et al., 2020).

Ascorbic acid (vitamin C) was measured using a microplate technique that reduces Fe^3+^ to Fe^2+^ using the reduced form of vitamin C (Stevens et al., 2006).

All these compounds were quantified using an external calibration based on commercially available pure standards (Sigma, Extrasynthese, or CaroteNature). In the absence of a pure standard, zeaxanthin palmitate could not be assayed with maximum confidence and it is therefore not studied in this article.

### 2.4 *In vitro* digestion and bioaccessibility assessment

Carotenoid bioaccessibility was measured on CI and DI fruits for goji and for tomato *H1311, M82, IL6-2*, and *IL12-4*, as they displayed the greatest differences in fruit carotenoid content (**Supplementary Figure 2**). Micellization efficiency, here described as bioaccessibility, was computed as the ratio between the compound quantity released from the food matrix and incorporated into mixed micelles and the compound quantity in the tested food. Carotenoid micellization was assessed using an *in vitro* digestion protocol including oral, gastric, and duodenal steps as previously described (Morand-Laffargue et al., 2023b) with the following modification: at the beginning of the protocol, 2 g of thawed tomato or goji fruit powder was placed in a 100-mL Erlenmeyer flask containing 32 mL of NaCl 0.9%. For goji, carboxyl ester lipase (CEL, Porcine pancreas, Sigma Aldrich, #26745) was added at the beginning of the duodenal digestion step, using 1 U mL^−1^ digestate, as suggested by a previous study (Wen et al., 2018). The CEL enzyme hydrolyzes numerous ester compounds, including the xanthophyll esters present in goji, and is known to be biologically active in the human digestive system (Chitchumroonchokchai and Failla, 2006). The rest of the protocol remained unchanged and was followed by centrifugation and 0.22-µm filtration (Millex-GS, mixed cellulose esters, Millipore) of the micellar supernatant. Aliquots were stored at –80°C in sealed tubes. The carotenoids in the micellar fractions of the digestates were extracted using slightly different protocols to those used for fresh fruit. For tomato, 500 µL of the micellar fraction was extracted with an ethanol–hexane mixture (1:4 v:v). The hexane phase of the extract was evaporated under nitrogen flow and then solubilized in a MeOH–DCM mixture before injection into HPLC-DAD as described elsewhere (Borel et al.; Morand-Laffargue et al., 2023a). For goji berry, 450 µL of the fraction was contacted with 30 mg of CaCO_3_ and 600 µL of hexane–ethanol (2:1 v:v) with butylated hydroxytoluene (BHT) (0.2%; w:v). After adding 300 µL of water, the two phases (hexane and aqueous) were separated by centrifugation. The hexane phase was placed in a microtube while 600 µL of 0.1% BHT hexane was added to the aqueous phase. A second centrifugation was used to recover the hexane phase and was added to the first. Between each step, a shaking step was performed. The two hexane phases were evaporated under nitrogen. The resulting dry extract was reconditioned immediately prior to analysis using UPLC-ESI-TQ by adding 150 µL of MTBE (methyl tert-butyl ether) followed by 300 µL of ethanol as described elsewhere (Dumont et al., 2020).

Micellar fractions from tomato digesta (100 µL) were injected into the HPLC-DAD as described elsewhere (Borel et al., 2021; Morand-Laffargue et al., 2023b). In our work, specific wavelengths were assigned for the quantification of all-trans-lycopene (472 nm), β-carotene and lutein (450 nm), phytoene (286 nm), and phytofluene (350 nm).

### 2.5 Statistical analysis and data processing

For tomato fruits, two-way analyses of variance (ANOVAs) were applied to test the impact of genotype (G), water treatment (T), and their interaction (G*T) (R software, version 4.2.2). Where at least one factor, or the interaction, was significant (*p* < 0.05), *post-hoc* pairwise comparisons were performed with Fisher’s least significant difference (LSD) test followed by a false discovery rate *p*-value adjustment using the Benjamini–Hochberg procedure (package “agricolae”, v 1.3-5). The impact of DI on goji fruits was evaluated independently using an unpaired two-tailed Student’s *t*-test. The standard deviations (SDs) of WUE and commercial fruit yield on a DW basis (CFDY) were calculated by considering error propagation formula for each product of the variables (Ku, 1966). For heatmaps, the Euclidean distance matrix was calculated and the complete-linkage clustering method (R, package “pheatmap”, v1.0.12) was applied, indicating significant comparisons with a “*” (*p* < 0.05). Plots were drawn using either R (package “ggplot2”, v3.4.0, and “ggprism”, v1.0.3) or GraphPad Prism (GraphPad Software, v9.0.0). Principal component analysis (PCA) of tomato carotenoids was carried out on centered and scaled data (R, package “ade4”, v.1.7-20) with individual values (95% confidence ellipse for each genotype-irrigation group) and variables plotted in the first two PC axes (R, package “factoextra”, v.1.0.7).

## 3 Results

### 3.1 Effects of water deficit on yield and water-use efficiency in tomato and goji

**Figure 1:**
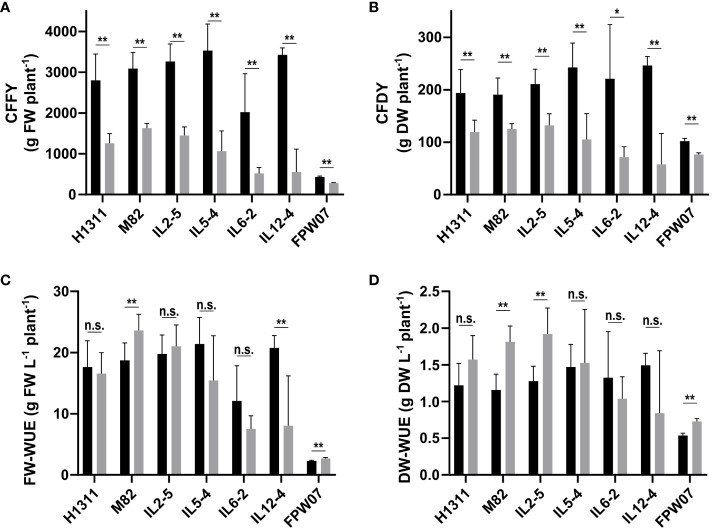
Cultivar and irrigation-related variability in commercial fruit yield expressed in **(A)** g fresh weight per plant (CFFY) and **(B)** g dry weight per plant (CFDY), and on water use efficiency expressed on a **(C)** fresh weight (FW-WUE) or **(D)** dry weight (DW-WUE) basis. (Black) Control Irrigation; (gray) Deficit Irrigation. Data confidence intervals are mean ± 95%. *N* = 6 plants for tomato genotypes (H1311, M82, IL2-5, IL5-4, IL-6-2, and IL12-4) and *N* = 3 averaged blocks of 10 plants for goji (FPW07). The statistical analysis was conducted using an unpaired Welch’s unequal variances *t*-test. In order to account for multiple comparisons and control the false discovery rate (FDR), the Benjamini and Hochberg procedure was employed to adjust p-values. N.s.: not significant. *p < 0.05, **p < 0.01.

Overall, DI and genotype had a strong impact on tomato production in terms of CFFY and CFDY (**Figure 1**). On average, the CFFY of CI plants ranged from 2,023 g plant^−1^ for IL6-2 to 3,531 g plant^−1^ for IL5-4, and for all six tomato genotypes, it fell by −64% under DI (from −47% for M82 to −84% for IL12-4). The average CFFY for goji plants was 433 g plant^−1^ under CI, but the average reduction under DI (−34%) was less severe than in tomato. Fruit DM content was higher under DI compared to CI: +14% on average for goji and from +26% (IL6-2) to +45% (IL12-4) in the tomato genotypes (**Table 1**). As a result, the average decrease of CFDY under DI ranged from −34% (M82) to −76% (IL12-4) for tomato and it was only −25% for goji.

**Table 1:** Characterization of tomato and goji berry fruit quality traits (mean ± SD, *N* = 5 analytical samples for tomato fruits and *N* = 6 samples for goji fruits, except for fruit weight, where *N* > 30 fruits for each genotype).

Genotype	H1311 CI	M82 CI	IL2-5 CI	IL5-4 CI	IL6-2 CI	IL12-4 CI	FPW07 CI
Fruit weight (g FW)	72.62 ± 8.3 a	50.33 ± 6.46 bc	35.06 ± 8.95 d	58.29 ± 3.52 b	43.88 ± 13.21 cd	75.81 ± 10.4 a	1.63 ± 0.25
Dry matter content (%)	6.92 ± 0.18 bc	6.17 ± 0.64 d	6.45 ± 0.23 cd	6.87 ± 0.33 bc	10.93 ± 0.54 a	7.19 ± 0.33 b	23.59 ± 0.43
Alcohol-insoluble matter (% DW)	38.91 ± 2.16 a	41.69 ± 5.54 a	46.82 ± 10.24 a	43.4 ± 5.47 a	37.42 ± 1.95 a	39.8 ± 2.88 a	4.16 ± 0.88
	Acids and sugars						
Glucose (g 100 g^−1^ DW)	15.61 ± 1.52 c	15.68 ± 1.78 c	18.13 ± 1.1 ab	16.75 ± 1.57 bc	20.08 ± 0.63 a	16.22 ± 0.83 bc	28.75 ± 1.93
Fructose (g 100 g^−1^ DW)	15.78 ± 1.47 d	18.34 ± 2.11 abc	19.37 ± 0.81 ab	17.69 ± 1.67 bcd	20.23 ± 0.63 a	17.02 ± 0.82 cd	28.81 ± 2.51
Sucrose (g 100 g^−1^ DW)	0.53 ± 0.11 bc	0.32 ± 0.02 d	0.44 ± 0.1 c	0.44 ± 0.06 c	0.65 ± 0.06 a	0.57 ± 0.04 ab	1.9 ± 0.45
Total sugars (g 100 g^−1^ DW)	31.93 ± 3.07 c	34.34 ± 3.89 bc	37.93 ± 1.82 ab	34.88 ± 3.27 bc	40.96 ± 1.25 a	33.81 ± 1.64 bc	59.46 ± 4.67
Citric acid (g 100 g^−1^ DW)	5.13 ± 0.48 b	6.75 ± 0.76 a	6.4 ± 0.82 a	6.1 ± 0.72 a	3.47 ± 0.19 c	6.87 ± 0.56 a	1.49 ± 0.25
Malic acid (g 100 g^−1^ DW)	0.32 ± 0.04 c	0.53 ± 0.06 a	0.41 ± 0.07 b	0.53 ± 0.07 a	0.24 ± 0.02 d	0.43 ± 0.04 b	0.28 ± 0.04
Total acids (g 100 g^−1^ DW)	5.45 ± 0.5 b	7.28 ± 0.81 a	6.81 ± 0.89 a	6.62 ± 0.76 a	3.71 ± 0.19 c	7.3 ± 0.6 a	1.77 ± 0.27
Sugar:acid ratio (%)	5.88 ± 0.58 b	4.72 ± 0.15 c	5.66 ± 0.91 b	5.28 ± 0.17 bc	11.07 ± 0.46 a	4.65 ± 0.3 c	34.13 ± 4.38
	Phenolics						
Rutin (mg kg^−1^ DW)	271.21 ± 87.6 a	248.55 ± 39.93 ab	202.96 ± 22.01 ab	224.56 ± 56.08 ab	163.06 ± 56.02 b	186.63 ± 24.27 ab	14.01 ± 1.44
Chlorogenic acid (mg kg^−1^ DW)	71.77 ± 11.44 bc	153.63 ± 32.5 a	190.52 ± 44.22 a	92.51 ± 17.35 b	105.33 ± 34.41 b	42.23 ± 11.45 c	16.02 ± 0.65
Naringenin chalcone (mg kg^−1^ DW)	79.95 ± 39.16 b	145.92 ± 49.58 a	184.35 ± 73.65 a	53.54 ± 16.91 bc	3.86 ± 7.04 c	19.53 ± 4.75 c	<L.Q.
Cryptochlorogenic acid (mg kg^−1^ DW)	39.74 ± 6.82 c	77.24 ± 19.99 a	76.98 ± 15.97 a	58.21 ± 9.87 b	74.81 ± 8.93 ab	24.58 ± 8.78 c	<L.Q.
Neochlorogenic acid (mg kg^−1^ DW)	-	-	-	-	-	-	11.44 ± 0.55
Total phenolics (mg kg^−1^ DW)	462.67 ± 100.27 b	625.35 ± 124.97 a	654.81 ± 86.99 a	428.82 ± 87.92 b	347.07 ± 56.82 bc	272.97 ± 17.52 c	41.46 ± 1.45
	Carotenoids						
Lycopene (mg kg^−1^ DW)	2824.95 ± 144.5 a	1561.83 ± 134.39 b	1685.65 ± 146.17 b	1694.02 ± 200.51 b	703.12 ± 50.29 c	864.57 ± 56.41 c	<L.Q.
Phytoene (mg kg^−1^ DW)	880.6 ± 109.43 b	564.13 ± 75.82 c	510.93 ± 103.74 c	533.42 ± 64.73 c	135.17 ± 6.79 d	1235.12 ± 82.66 a	<L.Q.
Phytofluene (mg kg^−1^ DW)	173.96 ± 17.22 a	87.36 ± 11.37 b	85.1 ± 16.81 b	86.09 ± 8.85 b	28.34 ± 3.02 c	167.07 ± 10.82 a	<L.Q.
β-carotene (mg kg^−1^ DW)	54.78 ± 6.02 b	43.41 ± 7.99 bc	45.8 ± 8.5 bc	39.9 ± 4.53 bc	375.08 ± 21.79 a	32.78 ± 3.34 c	3.51 ± 0.45
Lutein (mg kg^−1^ DW)	22.29 ± 2.06 a	15.11 ± 1.84 b	10.78 ± 2.13 c	10.7 ± 1.07 c	7.27 ± 0.55 d	9.36 ± 0.92 cd	<L.Q.
Zeaxanthin dipalmitate (mg kg^−1^ DW)	-	-	-	-	-	-	847.97 ± 57.99
Zeaxanthin (mg kg^−1^ DW)	-	-	-	-	-	-	4 ± 0.46
β-cryptoxanthin palmitate (mg kg^−1^ DW)	-	-	-	-	-	-	16.32 ± 1.61
β-cryptoxanthin (mg kg^−1^ DW)	-	-	-	-	-	-	<L.Q.
Total carotenoids (mg kg^−1^ DW)	3,956.57 ± 167.1 a	2,271.84 ± 228.71 b	2,338.26 ± 250.68 b	2,364.13 ± 264.14 b	1,248.98 ± 75.64 c	2,308.91 ± 149.34 b	871.8 ± 59.91
	Other health-related fruit properties						
Vitamin C (mg 100 g^−1^ DW)	320.69 ± 21.76 a	234.82 ± 23.12 b	238.95 ± 19.78 b	228.01 ± 25.11 b	196.28 ± 6.59 c	241.58 ± 12.79 b	121.92 ± 11.64
H-ORAC_FL_ (µmol TE g^−1^ DW)	17.01 ± 1.05 a	16.79 ± 3.37 a	15.49 ± 1.63 a	15.13 ± 1.92 a	13.18 ± 0.99 a	11.68 ± 1.88 a	18.30 ± 0.12

Excepting fruit weight and dry matter content, all traits are reported on a dry weight (DW) basis. Letters indicate significant differences between tomato genotypes (Fisher LSD followed by Benjamini–Hochberg p-value adjustment procedure). <L.Q. = below Limit of Quantification. - = not tested/not applicable.

Considering commercial fruits, tomato plants produced 18.4 g fresh mass L^−1^ water under CI and 15.4 g L^−1^ under DI. The relative variation under DI ranged from −61% (IL12-4) to +26% (M82) compared to the CI treatment. The FW-WUE was lower in goji than in tomatoes, with an average of 2.28 g fresh mass L^−1^ water for CI and a +19% increase under DI. On a dry mass basis (**Figures 1C, D**), tomato plants produced 1.32 g dry mass L^−1^ water under CI and the relative effect of DI ranged from −44% for IL12-4 to +57% for M82. Goji produced 0.54 g dry mass L^−1^ water with a +36% increase under DI compared to CI.

### 3.2 Impact of genotype and DI on the quality of tomato and goji fruits

Quality traits exhibited strong heterogeneity between tomato and goji, but also among tomato genotypes (**Table 1**). In tomato grown under CI conditions, the largest variations were observed for total carotenoid content with a 3.2-fold difference between the poorest (IL6-2) and richest (H1311) genotype. The sugar:acid ratio varied by a factor of 2.4 between the lowest (IL12-4) and highest (IL6-2) values. A 2-fold increase was observed between the lowest (IL6-2) and highest (M82) total acid contents and a 2.4-fold increase was observed between the lowest (IL12-4) and highest (IL2-5) total phenolic content. The high-lycopene cultivar H1311 also had the highest contents in phytofluene, lutein, ascorbic acid, and rutin. Under comparable conditions (CI), goji fruits displayed higher dry matter content, lower AIM content, and higher sugar and lower acid contents on a dry mass basis than ripe tomatoes, resulting in a higher sugar:acid ratio (34.1%). Goji fruits also displayed lower ascorbic acid (121.9 mg per 100 g^−1^ fruit DW) and lower hydrophilic antioxidant capacity (4.3 µmol TE per g^−1^ fruit DW) than tomatoes. The total phenolic and carotenoid contents on a dry mass basis were one order of magnitude higher in tomato fruit than in goji but values reported in **Table 1** are the sums of quantified compounds only, and thus they may be underestimated.

**Figure 2:**
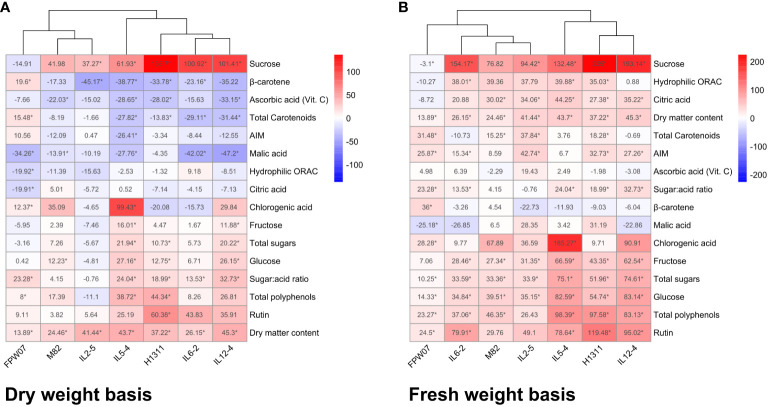
Water deficit effects on tomato and goji composition and composition-related traits (H-ORAC_FL_, DM) common between species. Values represent average relative effects of trait change between CI and DI fruits of each tomato and goji genotype on a dry weight basis **(A)** and fresh weight basis **(B)**. Significant changes are marked with an asterisk, *p* < 0.05.

The relative effect of DI on fruit traits is presented on the DW (**Figure 2A**) and FW (**Figure 2B**) basis. On a DW basis, DI had a negative or non-significant impact on total ascorbic acid, malic and citric acid, AIM, and hydrophilic antioxidant capacity in both tomato and goji fruits. DI had a negative or non-significant impact on total carotenoid and β-carotene content in tomato fruits, but a positive significant impact on the same compounds in goji. DI had a positive or insignificant impact on rutin, chlorogenic acid, total polyphenols, sugar:acid ratio, total sugars, glucose, fructose, and sucrose for both tomato and goji fruits. On an FW basis, DI had a positive or non-significant impact on 13 out of 16 measured traits for both tomato and goji genotypes. The exceptions were sucrose, which positively increased or was stable under DI for tomato but not for goji, β-carotene, which significantly increased in goji under DI but was stable for tomato, and malic acid, which significantly decreased for goji but was rather stable for tomato.

**Figure 3:**
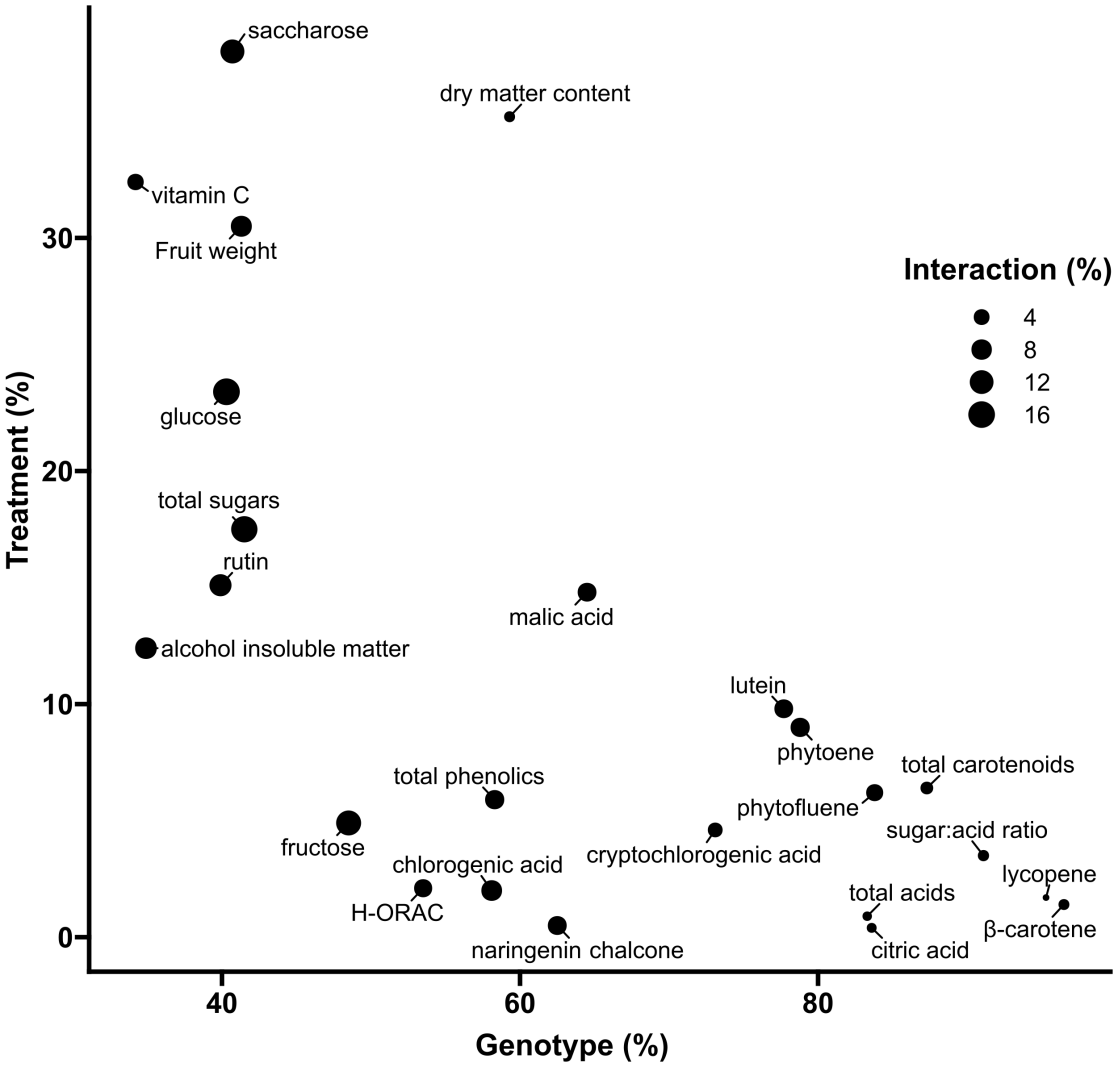
Two-way ANOVA variance decomposition of tomato fruits traits expressed on a dry weight basis. The *x*-axis and *y*-axis show the % of total variance (SSx/SStotal) accounted for by genotype and irrigation treatment, respectively. Symbol size represents the % of total variance accounted for by the interaction between genotype and treatment.

For all of the 24 measured fruit traits on a DW basis (**Figure 3**), univariate ANOVAs revealed a high proportion of variance explained by genotype (*p* < 0.001), ranging from 34.2% of total variance for ascorbic acid to 96.5% for β-carotene. The genotype factor accounted for 41.5% of total sugar variability, for 83.3% of total acid variability, for 91.1% of sugar:acid ratio, and for 87.3% of total carotenoid variability. Genotype variations in phenolic compounds ranged from 39.9% for rutin to 73.1% for cryptochlorogenic acid. The contribution of DI to the total variance of fruit metabolites ranged from 4.9% for fructose (*p* < 0.05) to 23.4% for glucose (*p* < 0.001) and 38% for sucrose (*p* < 0.001). It contributed to 32.4% of ascorbic acid variance (*p* < 0.001) and to less than 20% for all other compounds. Concerning carotenoids, DI significantly contributed (*p* < 0.001) from 1.4% for lycopene to 9.8% for lutein variance. With regard to the interactions between water treatment and genotypes, the highest contribution was observed for sugars, ranging from 12.3% for sucrose (*p* < 0.001) to 13.6% for fructose (*p* < 0.01) and 16.2% for glucose (*p* < 0.001) (**Figure 3**).

### 3.3 Effect of genotype and DI on micellar content and bioaccessibility of carotenoids in tomato fruits

**Figure 4:**
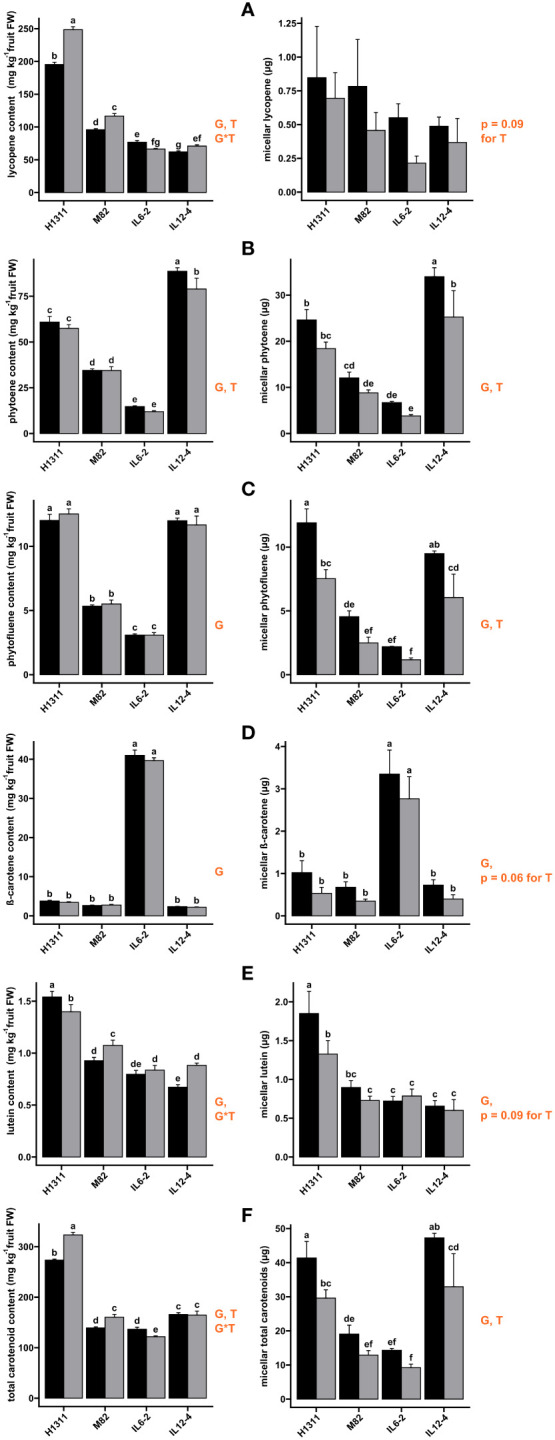
Effects of tomato genotype (G) and deficit irrigation (DI) on fruit carotenoid contents in fresh tomato fruits (left-hand panel) and in micelles (right-hand panel): lycopene **(A)**, β-carotene **(B)**, lutein **(C)**, phytoene **(D)**, phytofluene **(E)**, and total carotenoids **(F)**. (Black) Control Irrigation; (gray) Deficit Irrigation. Orange type indicates factor significance and sources of significant main effects: genotype **(G)**, deficit irrigation treatment (T), and interaction (G*T). Results are presented as mean ± SEM and letters indicate significant difference with *post-hoc* test Fischer LSD, followed by Benjamini-Hochberg false discovery rate *p*-value adjustment. Significant *p-*values are reported for α < 0.05.

The absolute quantities of carotenoids incorporated into mixed micelles after *in vitro* digestion are displayed in **Figure 4**. In tomatoes, the effect of genotype on fruit carotenoid content was not systematically reflected in the amount recovered in micelles due to variations in bioaccessibility (**Table 2**). For instance, more lycopene was observed in H1311 fresh fruits (+27.3%, *p* < 0.001) compared to other genotypes, but the total micellar lycopene recovered was independent of genotype (*p* = 0.26) as lycopene bioaccessibility was quite low (**Table 2**). Regarding the treatment effects, the micellar amounts of all carotenoids were reduced by DI compared to CI. This effect was significant and independent of genotype for phytoene (−30.3% on average) and phytofluene (−41.3%) recovery. Similarly, DI reduced, though not significantly, the micellar amount of lycopene (−36.4%, *p* = 0.09), β-carotene (−39.8%, *p* = 0.06), and lutein (−11.5%, *p* = 0.09) for all genotypes. When considering total carotenoids, a significant genotype effect (*p* < 0.001) was observed on the micellar carotenoid content, with a consistent (*p* = 0.61 for interaction) but negative DI impact (−31.7% on average, *p* < 0.01).

**Table 2:** Bioaccessibility of carotenoids from tomato (H1311, M82, IL6-2, and IL12-4) and goji (FPW07) fruits produced under Control Irrigation (CI) and Deficit Irrigation (DI) water treatments.

	H1311		M82		IL6-2		IL-12-4		FPW07	
	CI	DI	CI	DI	CI	DI	CI	DI	CI	DI
Lycopene	0.22 ± 0.17	0.14 ± 0.07 (−35.58%)	0.41 ± 0.36	0.2 ± 0.13 (−52.03%)	0.36 ± 0.14	0.16 ± 0.08 (−54.91%)	0.39 ± 0.11	0.26 ± 0.25 (−34.1%)	–	–
β-carotene	13.48 ± 7.58	7.72 ± 4.15 (−42.75%)	12.76 ± 5.61	6.29 ± 2.31 (−50.7%)	4.08 ± 1.58	3.49 ± 1.48 (−14.6%)	15.42 ± 6.17	8.98 ± 5.16 (−41.77%)	14.62 ± 2.47	14.08 ± 2.3 (−3.65%)
Lutein	60.05 ± 19.24	47.45 ± 13.42 (−20.97%)	48.35 ± 11.47	33.97 ± 7.15 (−29.74%)	45.32 ± 9.73 (%)	47.02 ± 13.23 (+3.74%)	48.78 ± 12.49	34.07 ± 17.66 (−30.15%)	–	–
Phytoene	20.23 ± 4.38	16.01 ± 2.72 (−20.84%)	17.47 ± 4.26	12.8 ± 2.87 (−26.75%)	22.69 ± 2.38	15.94 ± 3.21 (−29.75%)	19.17 ± 2.61	15.98 ± 8.57 (−16.63%)	–	–
Phytofluene	49.53 ± 8.99	30.06 ± 5.34 (−39.31%**)	42.57 ± 8.75	22.57 ± 9.76 (−6.98%**)	35.59 ± 2.6	18.97 ± 5.38 (−46.70%*)	39.58 ± 2.28	25.94 ± 16.05 (−34.47%*)	–	–
Zeaxanthin	–	–	–	–	–	–	–	–	0.41 ± 0.09	0.34 ± 0.12 (−17.7%)
β-cryptoxanthine	–	–	–	–	–	–	–	–	4.96 ± 0.5	3.34 ± 0.39 (−32.76%*)
Total fruit carotenoids	7.36 ± 0.96	4.41 ± 0.5 (−40.11%*)	6.8 ± 1.13	4 ± 0.65 (−41.23%*)	4.95 ± 0.57	3.59 ± 0.57 (−27.6%)	13.68 ± 1.39	9.92 ± 4.15 (−27.5%*)	0.56 ± 0.10	0.46 ± 0.12 (−16.70%)

Bioaccessibility was computed as the ratio between the compound quantity released from the food matrix and incorporated into mixed micelles after in vitro digestion and the compound quantity in the tested food. Results are mean ± SD. The relative change of carotenoid bioaccessibility between CI and DI fruits is reported in brackets, and significance levels are *p < 0.05; **p < 0.01.

These variations resulted from variations in bioaccessibility for each compound (**Table 2**). Lycopene bioaccessibility was reduced from −34.1% (IL12-4) to −54.9% (IL6-2) under DI (*p* = 0.03 for all genotype). The bioaccessibility of phytoene and phytofluene was higher than that of lycopene, but still a negative effect of DI was observed for all genotypes, ranging from −16.6% (IL12-4) to −29.8% (IL6-2) for phytoene and from −39.3% (H1311) to −47% (M82) for phytofluene. Similarly, lutein bioaccessibility was reduced (*p* < 0.05) by DI for all genotypes, from −21% (H1311) to −30.2% (IL12-4) with the exception of IL6-2 (+3.7%). The β-carotene bioaccessibility was significantly impacted by genotype and treatment (*p* < 0.01) without interaction between the two factors. DI reduced β-carotene bioaccessibility from −14.6% (IL6-2) to −50.7% (M82). IL6-2 fruits displayed the highest β-carotene content on an FW basis (**Figure 4**) but the lowest bioaccessibility value (4.1% for CI, **Table 2**). Regarding total carotenoid, the bioaccessibility, which averaged 8.2% for all CI fruit, significantly depended on genotype (*p* < 0.001) and it was reduced under DI (*p* < 0.001), from −27.5% (IL6-2) to −41.2% (M82). Across all tomato genotypes, carotenoid bioaccessibility under CI conditions can be ranked as follows: lutein (50.6%) > phytofluene (41.8%) > phytoene (19.9%) > β-carotene (11.4%) > lycopene (0.3%), while the ranking of the average relative impact of DI on carotenoid bioaccessibility is AS FOLLOWS: lutein (−19.3%) < phytoene (−23.5%) < β-carotene (−37.5%) < phytofluene (−41.9%) < lycopene (−44.2%).

### 3.4 Effect of DI on micellar content and bioaccessibility of carotenoids in goji berry

**Figure 5:**
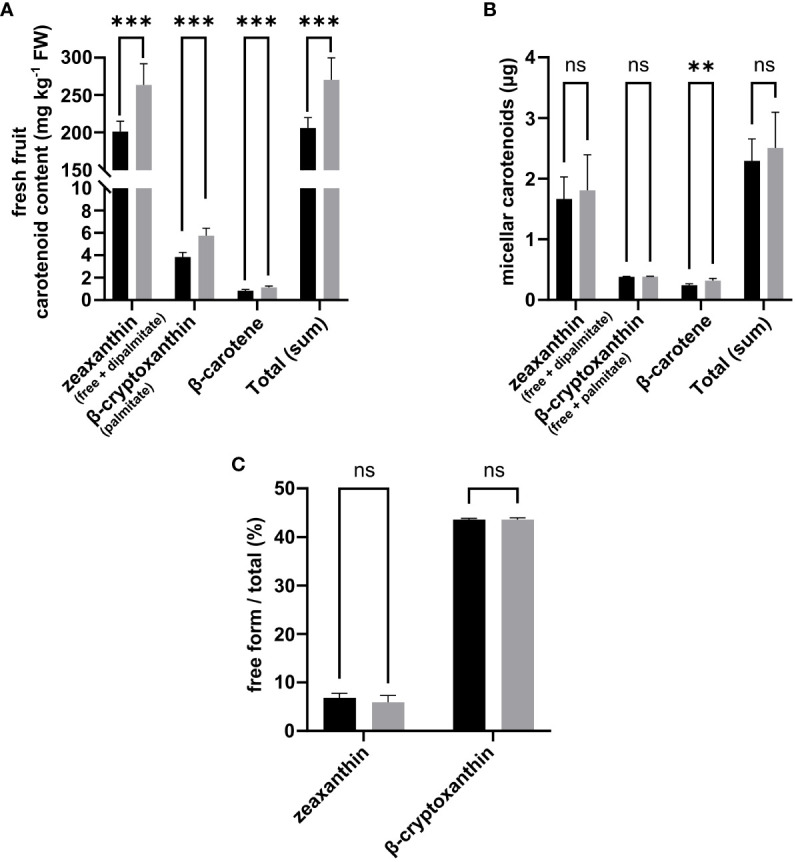
Effects of deficit irrigation on goji fruit (FPW07) carotenoid content on a fresh weight basis **(A)**, micellar carotenoids **(B)**, and fraction of carotenoid present in free form in the micellar phase **(C)**. Comparison between Control Irrigation (black) and Deficit Irrigation (gray) is performed for each compound using Student’s *t*-test with Benjamini–Hochberg *p*-value adjustment for multiple comparison. *N* = 6 samples for carotenoid contents in fresh fruit, *N* = 5 samples for *in vitro* digestion of micellar carotenoids.

As found by Hempel et al., the use of porcine CEL to cleave carotenoid esters in our protocol resulted in a relatively low percentage of free forms (<0.2%) in the digestate, raising questions over this enzyme’s ability to cleave carotenoid esters, although this is expected to occur at higher rates *in vivo* (Hui and Howles, 2002; Hempel et al., 2017 *a*). That said, a strong incorporation of free forms was observed in the micellar phase, averaging 100% of the free forms quantified at the end of the digestion phase for both zeaxanthin and β-cryptoxanthin (data not shown). Therefore, zeaxanthin and beta-cryptoxanthin in the fruit and micelles were expressed as the sum of the free and esterified forms. The fractions of free compound forms incorporated in the mixed micelles produced at the end of the *in vitro* digestion are shown in **Figure 5**.

On an FW basis, DI significantly increased the fresh fruit content in zeaxanthin (+ 31.1%, *p* < 0.001), β-cryptoxanthin (+49.4%, *p* < 0.001), and β-carotene (+36.1%, *p* < 0.001) compared to CI fruits (**Figure 5A**). Fresh fruit β-cryptoxanthin mainly accumulated in palmitate form with trace amounts of free β-cryptoxanthin in both CI and DI fruits (<0.6 µg g^−1^ fresh fruit weight) (**Figure 5**). No differences in zeaxanthin, β-cryptoxanthin, or total carotenoids were observed between CI and DI micellized fractions (**Figure 5B**). This was probably due to the reduction of the bioaccessibility of zeaxanthin (−17.7%, *p* = 0.33) and β-cryptoxanthin (−32.76%, *p* < 0.001) under DI (**Table 2**). Overall, while total carotenoids increased in fresh fruit under DI (+31.4%, *p* < 0.001), there was no difference in the total carotenoids recovered in mixed micelles between CI and DI samples because of a slight decrease in total carotenoid bioaccessibility under DI (−16.7%, *p* = 0.19) (**Figure 5B** and **Table 2**). With regard to deposition forms in the micellar phase, most of the zeaxanthin was recovered as dipalmitate, with an average of just 6.4% of total zeaxanthin being recovered as free zeaxanthin without any significant differences between CI and DI treatments. Most β-cryptoxanthin was recovered as palmitate but, compared to zeaxanthin, a higher fraction was recovered in free compound form, comprising, on average, 43.7% of total β-cryptoxanthin in the micellar phase, with no significant differences between CI and DI treatments (**Figure 5C**).

## 4 Discussion

The health benefit of fruit consumption is frequently emphasized, and the contributions of plant genetics and water deficit in modulating these effects are recognized. However, the results sometimes seem contradictory, because of the concomitant changes in both net accumulation (synthesis–catabolism) and concentration, as fruits accumulate less water under DI (Patanè et al., 2011; Génard et al., 2014; Petrović et al., 2019). Moreover, it may be a mistake to focus solely on the effects on fruit composition, since beyond the quantity ingested, the bioavailability of nutrients can make all the difference in terms of health effects. Indeed, to be successfully absorbed and metabolized in the human body, it is necessary for fruit micronutrients to be released from the food material and micellized in the small intestine. In the present study, these concentration–metabolism effects were further explored by focusing on compounds found in two plant species of the Solanaceae family, one of which has been little studied under DI conditions. The compositional analysis was also extended by investigating *in vitro* carotenoid bioaccessibility, an aspect that has been little examined so far under DI.

The present study clearly emphasized contrasted effects of DI on the accumulation and micellization of fruit carotenoids. In tomato, the richest fruits (H1311) did not always contain the most total micellar carotenoids, owing to substantial variation in micellization rates between different carotenoids (**Table 2**). The IL12-4 genotype, which contained significant amounts of highly bioaccessible carotenoid precursors such as phytoene and phytofluene, produced a quantity of total carotenoids incorporated in mixed micelles comparable to the H1311 genotype, while fresh fruits of IL12-4 contain almost half as many carotenoids as H1311 (**Table 1**). The very low bioaccessibility of lycopene (0.34% on average for all CI fruits, **Table 2**) was consistent with that reported in the literature for fresh fruit tomato digestion accompanied by a meal or with additional lipids—a 0.1% lycopene bioaccessibility from crude tomato was reported by Reboul et al. (2006), while Jeffery et al. (2012) reported 1.4% bioaccessibility. More generally, the present results showed an overall increased total carotenoid concentration (FW) under DI for both tomato and goji berries, but an overall limited incorporation of carotenoids into mixed micelles after digestion. DI concentrated some fruit compounds such as minerals and fibers, which may inhibit carotenoid incorporation in mixed micelles though matrix interactions (Ke et al., 2023). Zeaxanthin in its esterified form is the principal carotenoid (Hempel et al., 2017 *b*; Zhou et al., 2020) accumulated in goji berry; in the present work, the zeaxanthin dipalmitate content for cv. FPW07 ripe fresh berries was 200 mg kg^−1^ FW and 262 mg kg^−1^ FW, respectively, for CI and DI fruits, which is lower than that reported by Hempel et al. (2017*b*) (357 mg kg^−1^ FW) for goji mature fruits from unspecified cultivar, and lower than that reported by Zhou et al. (2020) (389 mg kg^−1^ FW) for cv. Zhongkelvchuan. While cv. FPW07 fruits remain an outstanding source of zeaxanthin, ranked above other zeaxanthin-rich foods, the low bioaccessibility of this compound in the applied *in vitro* digestion model and the esterified storage form of the compound would benefit from further comparison with other food matrices with a similar *in vitro* digestion protocol (Morand-Laffargue et al., 2023b). Numerous *in vitro* digestion protocols exist to evaluate food carotenoid bioaccessibility, for instance, the addition of yoghurt to raw fruit and vegetable (Jeffery et al., 2012) or the food supply with a standard meal composed of pureed potatoes, minced beef, and olive oil (Reboul et al., 2006). Such protocols improve carotenoid bioaccessibility through the additional presence of lipids. For goji, the inclusion of just 1% coconut oil fat increased the release into the digestive system of free zeaxanthin from 8% to 15%, and zeaxanthin dipalmitate from 27% to 44% (Hempel et al., 2017 *b*). Hempel et al. (2017*b*) evaluated the bioaccessibility of zeaxanthin and zeaxanthin dipalmitate at 6.7% and 2.3%, respectively, by *in vitro* digestion without any additional lipid or meal, but the foodstuff tested was dried goji fruits combined with water (1:1 w/w). This suggests that fruit drying could increase the bioaccessibility of goji berry carotenoids, by modifying the cell walls and enhancing carotenoid release. Such interactions between DI and fruit drying method deserve further attention.

The goji–tomato comparison in the present study intended to target two species of high nutritional value, one currently suffering from drought in production areas and the other offering a diversification alternative in the same regions. Although goji berry and tomato differ significantly in fruit size, cultivation method, and carotenoid content, similarities in biochemical changes and molecular regulation that underlie processes such as softening, color change, and ripening have been suggested (Cao et al., 2021). Our work confirmed that the FPW07 goji genotype offers a competitive alternative in regions affected by drought with a dry mass yield (CFDY) and water use efficiency (DW-WUE) similar to those of some tomato genotypes under DI. The expression of fruit health-related traits on an FW basis would qualify FPW07 fruits as “superconcentrates”. For example, average fresh weight vitamin C content was 28.8 mg kg^−1^ for goji fruits and 17.8 mg kg^−1^ for all tomato fruits under CI conditions. The same was observed for other health-related traits, such as hydrophilic antioxidant capacity (H-ORAC), with an average of 4.32 µmol Trolox eq. g fresh fruit^-1^ in goji fruits and 1.26 µmol Trolox eq. g fresh fruit^-1^ in all tomatoes under CI conditions. The H-ORAC_FL_ assay has been described as relevant to the quantification of the peroxyl radical scavenging capacity of food samples, but may not be adequate for the characterization of *in vivo* biological effects (Huang et al., 2005). The multiple antioxidant compounds present in fresh fruits could synergistically contribute to overall antioxidant activity. A comparative study between red (*L. barbarum*) and black (*L. ruthenicum*) goji fruits highlighted that the total antioxidant activities of the berry extracts significantly correlated to the total phenolic contents, but not to the total carotenoid contents (Islam et al., 2017). Goji berries contain a wide range of phenolics that could not be quantified in our experiment due to the absence of standards or availability of analytical procedures (Islam et al., 2017), and this test may account for their presence.

With regard to tomato fruit composition, the genetic effect was predominant on a dry matter basis (**Figure 3**) while the effect of DI was less than 20% of the total variance in all fruit traits. This may be due to the choice of genotypes studied, which focused on DI sensitivity and quality traits. Interestingly, the results only partially confirm the characteristics previously described for these tomato genotypes likely because of genotype by environment interactions. For instance, the drought resistance of IL2-5 is questionable in terms of yield (−30.6% reduction of average fruit mass under DI) as already suggested (Liu et al., 2018), whereas on a dry matter basis, IL2-5 fruits differed from other genotypes with a decreased sugar content, a different pattern of polyphenol change, a higher β-carotene level, but lower loss of total carotenoid under DI. Moreover, the higher antioxidants, ascorbic acid, and soluble solids previously reported in IL12-4 fruits (Sacco et al., 2013) may be due to the increase in fruit dry matter content (+16.5% in IL12-4 fruits compared to M82 under CI), and our data failed to characterize IL12-4 fruits as having improved “health-related” metabolite content. Thus, in line with other works, the beneficial impact of DI on fruit sugar, acid, and carotenoid contents, reported on an FW basis, mainly resulted from a concentration effect (less water accumulation in DI fruit) rather than a metabolic effect (increase of net accumulation of one metabolite) on fruits (Arbex de Castro Vilas Boas et al., 2017). Sucrose was, on average, the compound most positively affected by water deficit as already observed with other tomato genotypes (Petrović et al., 2019), but its concentration remained particularly low (**Table 1**). Regarding carotenoids, the lower content on a DW basis under DI suggested a higher catabolism. In particular, β-carotene can be broken down through non-enzymatic cleavage by reactive oxygen species (ROS) (Ramel et al., 2013) or enzymatic cleavage by carotenoid cleavage dioxygenase (CCD) enzymes, which are known to being produced towards the later stages of fruit maturation (Auldridge et al., 2006). Under DI, plants could be expected to produce higher amounts of abscisic acid (ABA), a β-carotene-derived major phytohormone involved in plant responses to abiotic stress (Mi et al., 2022).

In conclusion, it is necessary to carry out genetic breeding simultaneously on traits for adaptation to diminishing water resources and quality traits. It is also essential to look at the concentration of metabolites in the dry mass and not just in fresh fruit, and to go as far as measuring the bioavailability of micronutrients to assess potential effects on fruit health value. The overall low bioaccessibility of carotenoids suggests that simply increasing their concentration in fruit may not suffice to produce any significant enhancement of fruit health benefits. Species comparison is interesting in terms of metabolite profile and response to DI, which proved specific in the present study. Further investigations are needed to extend these results, for instance, by exploring more genetic resources and applying water deficit to specific periods of fruit development. It is also noteworthy that health-related metabolites such as phenolics or carotenoids are mainly located in the outer parts of the tomato fruit pericarp (Peng et al., 2008) so that investigating DI effects on each fruit tissue may be relevant.

## Data availability statement

The original contributions presented in the study are included in the article/**Supplementary Material**. Further inquiries can be directed to the corresponding author.

## Author contributions

TB: Conceptualization, Data curation, Formal Analysis, Investigation, Methodology, Writing – original draft. A-LF: Conceptualization, Investigation, Supervision, Writing – review & editing. DD: Investigation, Methodology, Writing – review & editing. CL: Investigation, Methodology, Writing – review & editing. CR: Investigation, Supervision, Writing – review & editing. PB: Methodology, Writing – review & editing. J-FL: Conceptualization, Funding acquisition, Investigation, Project administration, Supervision, Validation, Writing – review & editing.NB: Conceptualization, Funding acquisition, Investigation, Project administration, Supervision, Validation, Writing – review & editing. 

## References

[B1] Amagase H. Farnsworth N. R. (2011). A review of botanical characteristics, phytochemistry, clinical relevance in efficacy and safety of Lycium barbarum fruit (Goji). Food Res. Int. 44, 1702–1717. doi: 10.1016/j.foodres.2011.03.027

[B2] Arbex de Castro Vilas Boas A. Page D. Giovinazzo R. Bertin N. Fanciullino A. L. (2017). Combined effects of irrigation regime, genotype, and harvest stage determine tomato fruit quality and aptitude for processing into puree. Front. Plant Sci. 8. doi: 10.3389/fpls.2017.01725 PMC5633859 29051767

[B3] Auldridge M. E. McCarty D. R. Klee H. J. (2006). Plant carotenoid cleavage oxygenases and their apocarotenoid products. Curr. Opin. Plant Biol. 9, 315–321. doi: 10.1016/j.pbi.2006.03.005 16616608

[B4] Bassolino L. Petroni K. Polito A. Marinelli A. Azzini E. Ferrari M.. (2022). Does plant breeding for antioxidant-rich foods have an impact on human health? Antioxidants 11(4), 794. doi: 10.3390/antiox11040794 35453479 PMC9024522

[B5] Bertin N. Génard M. (2018). Tomato quality as influenced by preharvest factors. Scientia Hortic. 233, 264–276. doi: 10.1016/j.scienta.2018.01.056

[B6] Bohn T. Bonet M. L. Borel P. Keijer J. Landrier J. Milisav I.. (2021). Mechanistic aspects of carotenoid health benefits - Where are we now? Nutr. Res. Rev. 34(2), 276–302. doi: 10.1017/S0954422421000147 34057057

[B7] Borel P. (2003). Factors affecting intestinal absorption of highly lipophilic food microconstituents (Fat-soluble vitamins, carotenoids and phytosterols). Clin. Chem. Lab. Med. (CCLM) 41, 979–994. doi: 10.1515/CCLM.2003.151 12964802

[B8] Borel P. Hammaz F. Morand-Laffargue L. Creton B. Halimi C. Sabatier D.. (2021). Using black soldier fly larvae reared on fruits and vegetables waste as a sustainable dietary source of provitamin a carotenoids. Food Chem. 359, 129911. doi: 10.1016/j.foodchem.2021.129911 33951608

[B9] Cammarano D. Jamshidi S. Hoogenboom G. Ruane A. C. Niyogi D. Ronga D. (2022). Processing tomato production is expected to decrease by 2050 due to the projected increase in temperature. Nat. Food 3, 437–444. doi: 10.1038/s43016-022-00521-y 37118037

[B10] Cao Y. L. Li Y. L. Fan Y. F. Li Z. Yoshida K. Wang J. Y.. (2021). Wolfberry genomes and the evolution of Lycium (Solanaceae). Commun. Biol. 4(1), 671. doi: 10.1038/s42003-021-02152-8 34083720 PMC8175696

[B11] Chitchumroonchokchai C. Failla M. L. (2006). Hydrolysis of zeaxanthin esters by carboxyl ester lipase during digestion facilitates micellarization and uptake of the xanthophyll by Caco-2 human intestinal cells. J. Nutr. 136, 588–594. doi: 10.1093/jn/136.3.588 16484529

[B12] Costa J. M. Heuvelink E. P. (2018). "The global tomato industry", Tomatoes. (Wallingford UK: CABI), 1–26. doi: 10.1079/9781780641935.0001

[B13] Coyago-Cruz E. Corell M. Moriana A. Hernanz D. Stinco C. M. Mapelli-Brahm P.. (2022). Effect of regulated deficit irrigation on commercial quality parameters, carotenoids, phenolics and sugars of the black cherry tomato (Solanum lycopersicum L.) ‘Sunchocola’. J. Food Composition Anal. 105, 104220. doi: 10.1016/j.jfca.2021.104220

[B14] Diouf I. Derivot L. Koussevitzky S. Carretero Y. Bitton F. Moreau L.. (2020). Genetic basis of phenotypic plasticity and genotype × environment interactions in a multi-parental tomato population. J. Exp. Bot. 71, 5365–5376. doi: 10.1093/jxb/eraa265 32474596 PMC7501811

[B15] Djidonou D. Simonne A. H. Koch K. E. Brecht J. K. Zhao X. (2016). Nutritional quality of field-grown tomato fruit as affected by grafting with interspecific hybrid rootstocks. HortScience 51, 1618–1624. doi: 10.21273/HORTSCI11275-16

[B16] Dumont D. Danielato G. Chastellier A. Saint Oyant L. H. Fanciullino A. L. Lugan R. (2020). Multi-targeted metabolic profiling of carotenoids, phenolic compounds and primary metabolites in goji (Lycium spp.) berry and tomato (solanum lycopersicum) reveals inter and intra genus biomarkers. Metabolites 10, 1–17. doi: 10.3390/metabo10100422 PMC7589643 33096702

[B17] Eshed Y. Zamir D. (1994). A genomic library of Lycopersicon pennellii in L. esculentum: A tool for fine mapping of genes. Euphytica 79, 175–179. doi: 10.1007/BF00022516

[B18] Génard M. Baldazzi V. Gibon Y. (2014). Metabolic studies in plant organs: Don’t forget dilution by growth. Front. Plant Sci. 5, 1–5. doi: 10.3389/fpls.2014.00085 PMC3949113 24653732

[B19] Gilbert L. Alhagdow M. Nunes-Nesi A. Quemener B. Guillon F. Bouchet B.. (2009). GDP-d-mannose 3,5-epimerase (GME) plays a key role at the intersection of ascorbate and non-cellulosic cell-wall biosynthesis in tomato. Plant J. 60(30), 499–508. doi: 10.1111/j.1365-313X.2009.03972.x 19619161

[B20] Gomez L. Rubio E. Augé M. (2002). A new procedure for extraction and measurement of soluble sugars in ligneous plants. J. Sci. Food Agric. 82, 360–369. doi: 10.1002/jsfa.1046

[B21] Hempel J. Schädle C. N. Sprenger J. Heller A. Carle R. Schweiggert R. M. (2017). Ultrastructural deposition forms and bioaccessibility of carotenoids and carotenoid esters from goji berries (Lycium barbarum L.). Food Chem. 218, 525–533. doi: 10.1016/j.foodchem.2016.09.065 27719945

[B22] Huang D. Boxin O. U. Prior R. L. (2005). The chemistry behind antioxidant capacity assays. J. Agric. Food Chem. 53, 1841–1856. doi: 10.1021/jf030723c 15769103

[B23] Huang D. Ou B. Hampsch-Woodill M. Flanagan J. A. Prior R. L. (2002). High-throughput assay of oxygen radical absorbance capacity (ORAC) using a multichannel liquid handling system coupled with a microplate fluorescence reader in 96-well format. J. Agric. Food Chem. 50, 4437–4444. doi: 10.1021/jf0201529 12137457

[B24] Hui D. Y. Howles P. N. (2002). Carboxyl ester lipase: Structure-function relationship and physiological role in lipoprotein metabolism and atherosclerosis. J. Lipid Res. 43, 2017–2030. doi: 10.1194/jlr.R200013-JLR200 12454261

[B25] Islam T. Yu X. Badwal T. S. Xu B. (2017). Comparative studies on phenolic profiles, antioxidant capacities and carotenoid contents of red goji berry (Lycium barbarum) and black goji berry (Lycium ruthenicum). Chem. Cent. J. 11, 1–8. doi: 10.1186/s13065-017-0287-z 29086843 PMC5483215

[B26] Jeffery J. L. Turner N. D. King S. R. (2012). Carotenoid bioaccessibility from nine raw carotenoid-storing fruits and vegetables using an in *vitro* model. J. Sci. Food Agric. 92, 2603–2610. doi: 10.1002/jsfa.5768 22806183

[B27] Jiao Y. Liu G. (2020). Goji berry: a novel nutraceutical ‘Superfruit’ for florida master gardeners. Edis. 2020. doi: 10.32473/edis-hs1391-2020

[B28] Jordan M. O. Sauge M. H. Vercambre G. (2020). Chemical and growth traits of the peach tree may induce higher infestation rates of the green peach aphid, Myzus persicae (Sulzer). Pest Manage. Sci. 76, 797–806. doi: 10.1002/ps.5583 31400056

[B29] Ke Y. Deng L. Dai T. Xiao M. Chen M. Liang R.. (2023). Effects of cell wall polysaccharides on the bioaccessibility of carotenoids, polyphenols, and minerals: an overview. Crit Rev Food Sci Nutr 63 (32), 11385–11398. doi: 10.1080/10408398.2022.2089626 35730204

[B30] Ku H. H. (1966). Notes on the use of propagation of error formulas. J. Res. Natl. Bureau Standards Section C: Eng. Instrumentation 70C 263, 263–273. doi: 10.6028/jres.070c.025

[B31] Liu Y.-S. Gur A. Ronen G. Causse M. Damidaux R. Buret M.. (2003). There is more to tomato fruit colour than candidate carotenoid genes. Plant Biotechnol. J. 1, 195–207. doi: 10.1046/j.1467-7652.2003.00018.x 17156032

[B32] Liu M. Yu H. Zhao G. Huang Q. Lu Y. Ouyang B. (2017). Profiling of drought-responsive microRNA and mRNA in tomato using high-throughput sequencing. BMC Genomics 18, 1–18. doi: 10.1186/s12864-017-3869-1 28651543 PMC5485680

[B33] Liu M. Yu H. Zhao G. Huang Q. Lu Y. Ouyang B. (2018). Identification of drought-responsive microRNAs in tomato using high-throughput sequencing. Funct. Integr. Genomics 18, 67–78. doi: 10.1007/s10142-017-0575-7 28956210

[B34] Matsumoto C. Yada H. Hayakawa C. Hoshino K. Hirai H. Kato K.. (2021). Physiological characterization of tomato introgression line IL5-4 that increases brix and blossom-end rot in ripening fruit. Horticulture J. 90, 215–222. doi: 10.2503/hortj.UTD-264

[B35] Mi J. Vallarino J. G. Petřík I. Novák O. Correa S. M. Chodasiewicz M.. (2022). A manipulation of carotenoid metabolism influence biomass partitioning and fitness in tomato. Metab. Eng. 70, 166–180. doi: 10.1016/j.ymben.2022.01.004 35031492

[B36] Morand-Laffargue L. Creton B. Halimi C. Sabatier D. Desmarchelier C. Borel P. (2023a). Black soldier fly larvae as a sustainable and concentrated source of bioavailable lutein for feed. J. Insects Food Feed. doi: 10.1163/23524588-20230107

[B37] Morand-Laffargue L. Hirschberg J. Halimi C. Desmarchelier C. Borel P. (2023b). The zeaxanthin present in a tomato line rich in this carotenoid is as bioavailable as that present in the food sources richest in this xanthophyll. Food Res. Int. 168, 112751. doi: 10.1016/j.foodres.2023.112751 37120204

[B38] Morelli L. Rodriguez-Concepcion M. (2023). Open avenues for carotenoid biofortification of plant tissues. Plant Commun. 4, 100466. doi: 10.1016/j.xplc.2022.100466 36303429 PMC9860184

[B39] Ozminkowski R. H. (2018). U.S. Patent No. 9,861,046 Washington, DC: U.S. Patent and Trademark Office.

[B40] Patanè C. Tringali S. Sortino O. (2011). Effects of deficit irrigation on biomass, yield, water productivity and fruit quality of processing tomato under semi-arid Mediterranean climate conditions. Scientia Hortic. 129, 590–596. doi: 10.1016/j.scienta.2011.04.030

[B41] Peng Y. Zhang Y. Ye J. (2008). Determination of phenolic compounds and ascorbic acid in different fractions of tomato by capillary electrophoresis with electrochemical detection. J. Agric. Food Chem. 56, 1838–1844. doi: 10.1021/jf0727544 18284201

[B42] Petrović I. Savić S. Jovanović Z. Stikić R. Brunel B. Sérino S.. (2019). Fruit quality of cherry and large fruited tomato genotypes as influenced by water deficit. Zemdirbyste 106, 123–128. doi: 10.13080/z-a.2019.106.016

[B43] Poggioni L. Romi M. Guarnieri M. Cai G. Cantini C. (2022). Nutraceutical profile of goji (Lycium barbarum L.) berries in relation to environmental conditions and harvesting period. Food Biosci. 49, 101954. doi: 10.1016/j.fbio.2022.101954

[B44] Poiroux-Gonord F. Bidel L. P. R. Fanciullino A. L. Gautier H. Lauri-Lopez F. Urban L. (2010). Health benefits of vitamins and secondary metabolites of fruits and vegetables and prospects to increase their concentrations by agronomic approaches. J. Agric. Food Chem. 58, 12065–12082. doi: 10.1021/jf1037745 21067179

[B45] Ramel F. Mialoundama A. S. Havaux M. (2013). Nonenzymic carotenoid oxidation and photooxidative stress signalling in plants. J. Exp. Bot. 64, 799–805. doi: 10.1093/jxb/ers223 22915744

[B46] Reboul E. Richelle M. Perrot E. Desmoulins-Malezet C. Pirisi V. Borel P. (2006). Bioaccessibility of carotenoids and vitamin E from their main dietary sources. J. Agric. Food Chem. 54, 8749–8755. doi: 10.1021/jf061818s 17090117

[B47] Ripoll J. Urban L. Staudt M. Lopez-Lauri F. Bidel L. P. R. Bertin N. (2014). Water shortage and quality of fleshy fruits-making the most of the unavoidable. J. Exp. Bot. 65, 4097–4117. doi: 10.1093/jxb/eru197 24821951

[B48] Ronen G. Carmel-Goren L. Zamir D. Hirschberg J. (2000). An alternative pathway to β-carotene formation in plant chromoplasts discovered by map-based cloning of Beta and old-gold color mutations in tomato. Proc. Natl. Acad. Sci. United States America 97, 11102–11107. doi: 10.1073/pnas.190177497 PMC27155 10995464

[B49] Rousseaux M. C. Jones C. M. Adams D. Chetelat R. Bennett A. Powell A. (2005). QTL analysis of fruit antioxidants in tomato using Lycopersicon pennellii introgression lines. Theor. Appl. Genet. 111, 1396–1408. doi: 10.1007/s00122-005-0071-7 16177901

[B50] Sacco A. Di Matteo A. Lombardi N. Trotta N. Punzo B. Mari A.. (2013). Quantitative trait loci pyramiding for fruit quality traits in tomato. Mol. Breed. 31, 217–222. doi: 10.1007/s11032-012-9763-2 23316114 PMC3538004

[B51] Sérino S. Gomez L. Costagliola G. U. Y. Gautier H. (2009). HPLC assay of tomato carotenoids: validation of a rapid microextraction technique. J. Agric. Food Chem. 57, 8753–8760. doi: 10.1021/jf902113n 19769393

[B52] Slavin J. L. Lloyd B. (2012). Health Bene fi ts of Fruits and Vegetables. Adv. Nutr. 3 (4), 506–516. doi: 10.3945/an.112.002154 22797986 PMC3649719

[B53] Socaciu C. (2007). Food colorants: chemical and functional properties (Chemical & Functional properties of food components) 3–21. doi: 10.1201/9781420009286

[B54] Spinoni J. Vogt J. V. Naumann G. Barbosa P. Dosio A. (2018). Will drought events become more frequent and severe in Europe? Int. J. Climatology 38, 1718–1736. doi: 10.1002/joc.5291

[B55] Stevens R. Buret M. Garchery C. Carretero Y. Causse M. (2006). Technique for rapid, small-scale analysis of vitamin C levels in fruit and application to a tomato mutant collection. J. Agric. Food Chem. 54, 6159–6165. doi: 10.1021/jf061241e 16910702

[B56] Story E. N. Kopec R. E. Schwartz S. J. Keith Harris G. (2010). An update on the health effects of tomato lycopene. Annu. Rev. Food Sci. Technol. 1, 189–210. doi: 10.1146/annurev.food.102308.124120 22129335 PMC3850026

[B57] Van Het Hof K. H. West C. E. Weststrate J. A. Hautvast J. G. A. J. (2000). Dietary factors that affect the bioavailability of carotenoids. J. Nutr. 130, 503–506. doi: 10.1093/jn/130.3.503 10702576

[B58] Wang W. (2019). U.S. Patent Application No. 15/932,142.

[B59] Wei Y. Xu X. Tao H. Wang P. (2006). Growth performance and physiological response in the halophyte Lycium barbarum grown at salt-affected soil. Ann. Appl. Biol. 149, 263–269. doi: 10.1111/j.1744-7348.2006.00092.x

[B60] Wen X. Hempel J. Schweiggert R. M. Wang Y. Ni Y. Carle R. (2018). Screening of critical factors influencing the efficient hydrolysis of zeaxanthin dipalmitate in an adapted in *vitro*- digestion model. Food Chem. 257, 36–43. doi: 10.1016/j.foodchem.2018.02.116 29622222

[B61] Wu B. H. Génard M. Lescourret F. Gomez L. Li S. H. (2002). Influence of assimilate and water supply on seasonal variation of acids in peach (cv Suncrest). J. Sci. Food Agric. 82, 1829–1836. doi: 10.1002/jsfa.1267

[B62] Zhou Y. Lai Y. Chen Z. Qu H. Ma S. Wang Y.. (2020). Evolution of physiological characteristics and nutritional quality in fresh goji berry (Lycium barbarum) stored under different temperatures. J. Food Process. Preservation 44, 1–12. doi: 10.1111/jfpp.14835

