# Supplementary material for: Effect of long-term deficit irrigation on tomato and goji berry quality: from fruit composition to in vitro bioaccessibility of carotenoids
Source: Front Plant Sci. 2024 Jan 24;15:1339536. doi: 10.3389/fpls.2024.1339536 (PMC10847359; doi:10.3389/fpls.2024.1339536)

Supplementary Figure S1. Changes through time in the volumetric water content of tomato (left) and goji berry plants (right) during the experimental period for "Control Irrigation" and "Deficit Irrigation" treatments (smoothed mean +/- SD, N = 5 probes/species/treatment).

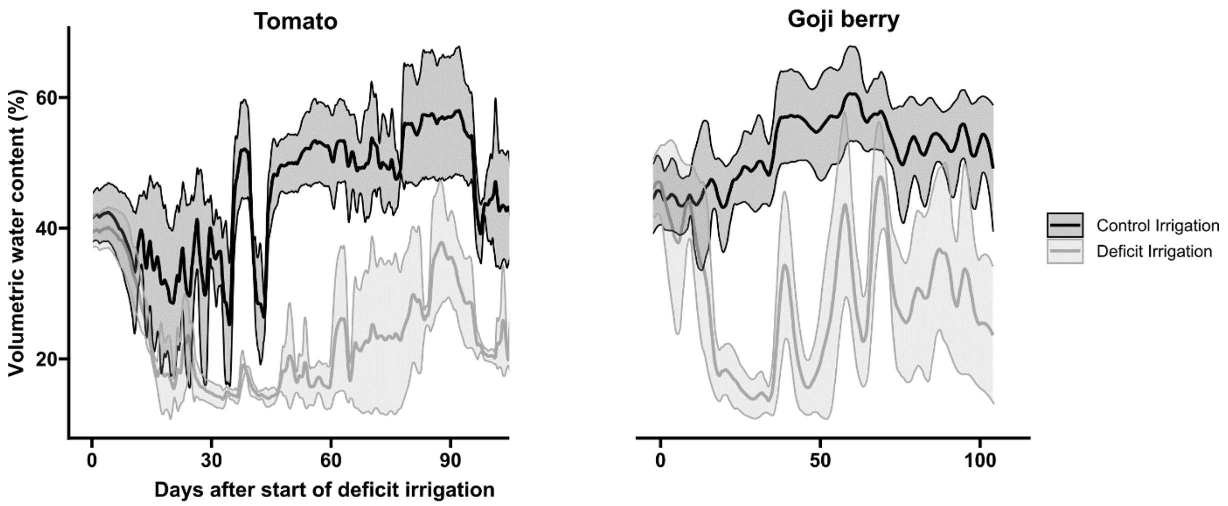

Supplementary Figure S2. Biplot for the two first components of the principal component analysis of tomato carotenoid concentrations on a dry weight basis. N = 5 samples for each group, labelled by genotype (H1311, M82, IL2-5, IL5-4, IL6-2, IL12-4) and water treatment = Control Irrigation (CI) or Deficit Irrigation (DI). M82 was used as a genetic reference for the other ILs Ellipses represent 95% confidence intervals.

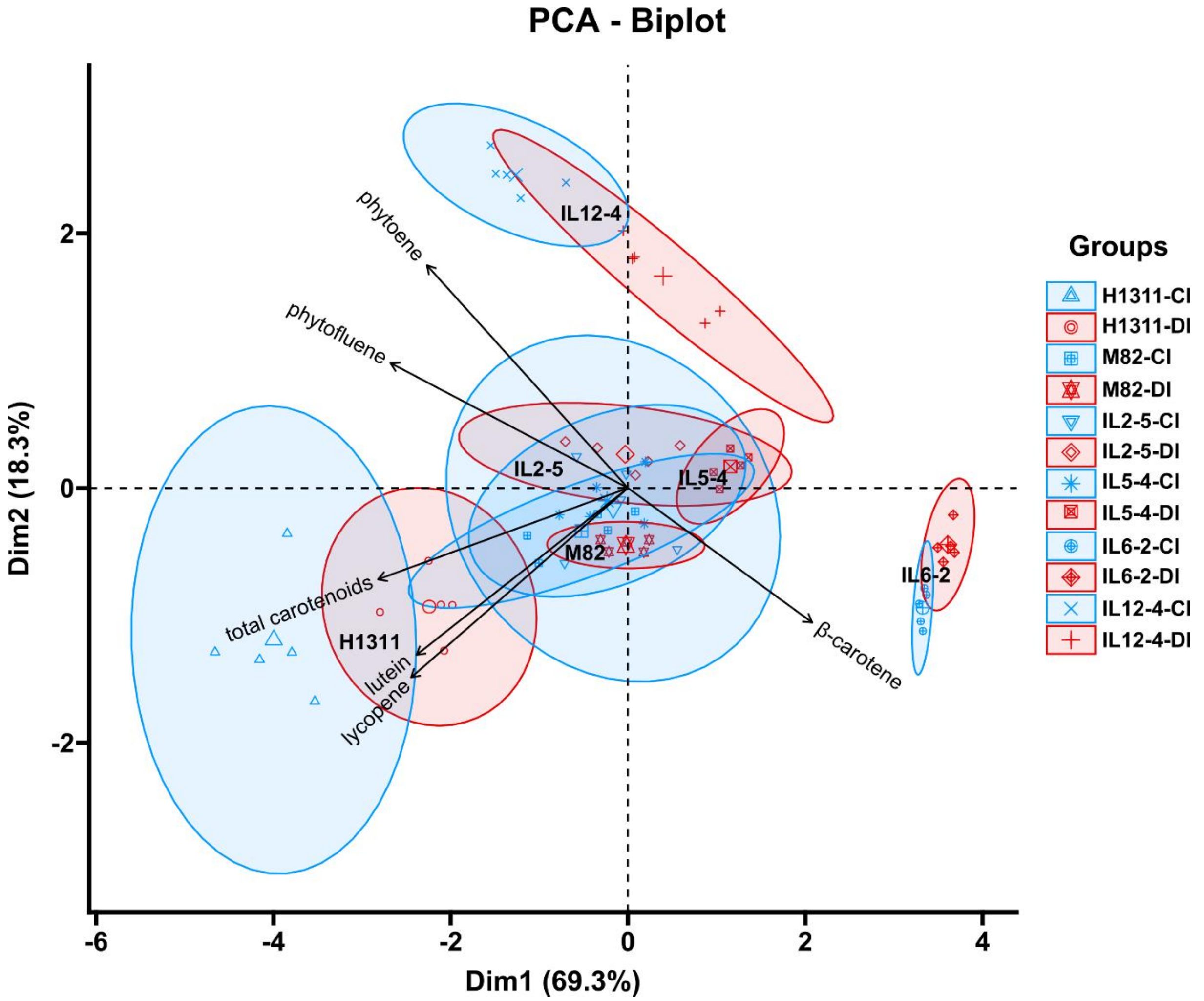

Supplement: Supplementary file 1 [file DataSheet_1.pdf]
